# Analysis of the current state of frailty indexes and their implementation for aging intervention studies

**DOI:** 10.18632/aging.206307

**Published:** 2025-08-26

**Authors:** Oliver G. Frost, Anna Barkovskaya, Michael J. Rae, Marcela Atzori, Abdelhadi Rebbaa, Amit Sharma

**Affiliations:** 1 Centre for Biological Engineering, School of Mechanical, Electrical and Manufacturing Engineering, Loughborough University, Loughborough LE11 3TU, UK; 2 Lifespan Research Institute, Mountain View, CA 94041, USA

**Keywords:** frailty, rodents, frailty index, phenotype, aging

## Abstract

Animal lifespan studies are foundational to developing interventions against the biological aging process. In recent years, there has been rising interest in characterizing the effects of longevity therapeutics on health span. Frailty indexes, originally developed to assess clinical frailty in aging humans, have shown promise as measurements of biological age and have been adopted for use in rodent aging biology. This Perspective looks at the current state of rodent frailty indexes and how they are implemented. The differences in frailty parameters used to calculate these indexes have led to inconsistencies between studies defining frailty. In this Perspective, we have highlighted those differences and made recommendations for implementing protocols for frailty index measurement.

## INTRODUCTION

Biological aging is the accumulation of damage to the cellular and molecular functional units of tissues over time, leading to breakdowns of physiological systems, age-related disease, and, ultimately, mortality [1]. This process, combined with the global population undergoing a demographic transition in which a rising percentage is living into historically advanced ages, motivates the search for therapies that would intervene in the biological aging process and thus delay, decelerate, or reverse multiple manifestations of age-related ill health [2].

The cornerstone test for longevity therapeutics in animal models is the lifespan study, emphasizing maximum lifespan [3, 4]. However, potential treatments should also increase the time spent in good health, motivating interest in developing quantitative metrics for health span. One approach to this metric is the rodent frailty index (FI). In humans, frailty is a clinical syndrome characterized by a decline in physiological resilience and increased vulnerability to adverse health outcomes, particularly in the elderly [5]. As a population ages, there is an increase in frailty, especially over the age of 65, with a significant correlation between frailty, mortality, and hospitalization being evidenced [6, 7]. Hence, researchers have constructed practical and objective measures of frailty to manage and test new interventions against the syndrome. The frailty index is a quantitative, multidimensional approach that evaluates an individual’s level of frailty based on the accumulation of health deficits [8]. Subsequent research has shown that FIs are robust predictors of mortality in humans, even in people early in middle age who are not colloquially “frail” [9, 10] and similar deficit-based constructs respond to putative longevity interventions in humans [10–13].

More recently, rodent FIs have been developed on principles like the human originals, using a panel of physical and/or clinical parameters to measure frailty. However, developing FIs in rodents is not straightforward. Researchers have adopted different approaches by varying the types and numbers of parameters to be included in the FI and cut-off points that classify an animal as frail. This variation results in each study having a different operational definition of frailty, which reduces the ability to compare findings across studies, thereby impeding progress toward longevity therapeutics. Even in humans, where sample sizes are larger and clinical data for FI construction is broader and more robust, different frailty instruments vary in which patients they identify as frail and are challenging to implement in clinical settings [14].

This Perspective highlights these differences and compares rodent and human frailty index scores. We summarise the published strategies for implementing frailty indexes, discuss their strengths and weaknesses, and implement a frailty index based on physical performance with our young and old mice. The Perspective concludes by making recommendations regarding FI use for studies of aging, age-related disease, and longevity therapeutics.

## MATERIALS AND METHODS

### Animals

Young (3-4 months), middle-aged (18 months), and old (28 months), mainly male C57BL/6 mice, were acquired from Charles River Laboratories (Wilmington, MA, USA) (n = 3-7 per group). Young 3-month-old (n=5) mice were all male, 4-month-old mice included two males and two females, 18-month-old (n=5) were all male, and 28-month-old (n=4) were three males and one female. Mice were housed in a vivarium with *ad libitum* access to food (Teklad Global Soy protein-free, Envigo) and water (Aquavive Water, Innovive Inc). Cages were changed every 1-2 weeks depending on the number per cage, and documents were maintained under national and local regulations. All animal experiments in this study are approved by the Lifespan Research Institute IACUC committee following the “Guide for the Care and Use of Laboratory Animals” prepared by the Institute for Laboratory Animal Research, National Academy of Sciences.

### Grip strength

C57BL/6 mice were used to establish baseline grip strength using the Grip Strength Meter (47200, Ugo Basile®). The mice were held by the mid-base of the tail, allowing their front paws to grip the bar. Once gripped, the mice were pulled back steadily, keeping them horizontal. The flat bar was used to measure the highest forelimb grip strength of the mice. To account for weight increases in older mice, results were normalised to weight. Each mouse was tested 4 times. The weakest measurement was removed and the average of the remaining scores was calculated.

### Open field testing

C57BL/6 mice were used to establish a baseline of mobility and behavior using open-field testing (Noldus Ethovision XT 17.5). Two arenas side by side (50x50cm) were set up with Basler GenICam (acA1300-60, Basler) above. Arena settings were utilized to establish each arena's scale (50cm) and center and periphery. The periphery is defined as the outermost 10cm on either side of the center (30cm) of the arena. Each mouse began the experiment in the bottom left corner and was recorded for 10 minutes. Video analysis software (Ethovision XT 17.5) was used to track the mice using the center body point and calculate the total distance moved, percent of time spent in movement, average velocity, body elongation, and mobility. Between each trial, 70% ethanol was used to clean the arena to remove scent marks, and a 10-minute wait period was used to allow ethanol evaporation.

### Measures used to construct the frailty index (FI)

The factors from open-field testing used to comprise the frailty index are previously described [15, 16]. They include the total distance moved (tracked by center-point over 10 minutes in cm), the maximum distance between two consecutive points in the tracks in cm (available in trial statistics of distance moved in the Ethovision analysis profile), total duration of movement (s), proportion of time spent moving (%), meander (change in direction per unit distance moved measured in degrees/cm from 0°-180°), average velocity (cm/s), rearing frequency (occurrence/time), and weight (g). For both studies, the duration of the open-field test was 10 minutes.

### Frailty index cutoff points

The young, middle-aged, and old mice required reference values to determine frailty levels. For these, we included our 3-4-month-old mice and the reference values from two previous reports, which were in one case males aged 5 months (n = 5) [15], and in the other males and females aged 13.5 months (n=3 per sex) [16]. When using reference values from 3-month-old male mice, all females from our cohort were removed. For each mouse, each factor of the FI was compared to the reference values. The frailty scores were applied if the measured score differed from the reference by at least 1SD (Standard Deviation). They were graded as follows: less than 1SD scored 0; values differed by ± 1 SD scored 0.25; values that differed by ± 2 SD scored 0.5; values differed by ± 3SD scored 0.75; values that differed by more than 3SD scored maximum frailty of 1. The sum of these scores for each factor was divided by the number of parameters (8) to produce a total frailty score for each mouse [16].

### Statistics

GraphPad Prism V10.1.0 software was used to perform statistical analysis. A two-way ANOVA analysis was used to see if two or more independent variables affected the dependent variable (multiple age groups in frailty scores or grip strength). To be considered statistically significant, the P-value must be ≤ 0.05 and was quantified in the following order. * = p ≤ 0.05, ** p = ≤ 0.01, *** = p ≤0.001, **** = p ≤0.0001. For each experiment and each condition, n ≥ 3.

### Study selection process

A rigorous literature review with a range of keywords was used to search peer-reviewed journals: frailty, frailty index, aging, mice, longevity, healthspan, rodents, and phenotype. Databases searched included PubMed, EBSCOhost, Google Scholar, and Loughborough University Ex Libris. Only original research articles were included in the criteria, literature reviews and other article types were excluded. Only frailty indexes based on rodents were included with human frailty indexes excluded. This method provided 18 peer-reviewed articles published between 2012 and 2023. Other papers were identified but not included as this Perspective focuses on frailty indexes that are novel, modified, or further validate established versions [15–32].

## RESULTS

All FIs included in this review provide a numeric score indicating the level of frailty present, but their respective methods differ significantly (Supplementary Table 1). When choosing which established FI to implement, one must examine what cutoff point to utilize, what reference values to use if a scaled frailty score is desired or the cohort is small, the equipment available, and the factors to include. For instance, scoring systems may rely on quantifying physical performance (13/18 studies) [15, 16, 19, 22–26, 28, 29, 31–33] or clinical observations (13/18 studies) [15, 16, 18–22, 25, 27, 29–32], with several articles using both [15, 16, 19, 22, 25, 29, 31, 32]. Those measuring physical fitness are modelled on Fried et al., 2001, who created a human frailty index measuring four key factors: weakness, slowness, low activity, and poor endurance [34].

The contents of the FIs vary significantly, depending on whether clinical observations or physical outputs are measured. For instance, those focusing on physical measurements have fewer items in their FI (4-5 items (7/18 studies) [17, 22–24, 26, 28, 31] or 8 items (4/18 studies)) [15, 16, 29, 32], whereas those using clinical observations measured 23-34 items (12/18 studies) [15, 16, 18–21, 25, 27, 29–32]. Additionally, there is variation in the cutoff points used to determine whether a subject is frail and to what extent. This includes 0.8SD from a reference point or the lowest 20% of a cohort (7/18 studies) [22–24, 26, 28, 29, 31], 1.5SD (4/18 studies) [17, 23, 27, 31], a staggered cutoff point of 1, 2, 3, 3+SD (9/18 studies) [15, 16, 18–21, 25, 27, 30], or visual determination of 0 = not frail, 0.5 = mildly frail, and 1 = frail (9/18 studies) [15, 18–21, 27, 29, 30, 32]. The cohort mean is often utilized as the reference point. But if a staggered cutoff point is used, a reference value is required from a control subject group. If the cohort mean-SD is used as the cut-off point, the frailty scoring is binary, either not frail = 0 or frail = 1. 0. When multiple cut-off points are used (1, 2, 3, 3+SD), then frailty is scored in a gradient (0, 0.25, 0.5, 0.75, 1).

While human frailty is the basis for rodent FIs, how to compare data from rodent FIs to equivalent effects if translated to humans is not clear. One approach is to compare deficits among animals and humans of similar biological age. Various investigators have developed systematic methods of equating biological age between mice and humans, including development, epigenetic age clocks, gene expression patterns, disease onset ages, median and maximum lifespan proportions, and/or the trajectory of the survival curve [35–38]. As a result, several studies [15–17, 20–22, 26, 27] have linked their rodent analysis to quantitative human data, whereas others make no direct comparison (Supplementary Table 1) [18, 19, 23–25, 28–32].

Those making such comparisons often use deficit accumulation as the key metric (natural log of FI vs. age) after normalizing human and mouse data sets to 90% mortality values or comparing the equivalent ages of the two species. The problem with using corresponding ages is the inconsistency across the literature in what age cutoffs are considered equivalent. Liu et al. identified 9% of 27-28-month-old mice as frail, consistent with frailty levels in 80-year-old humans [17]. While Baumann et al. found all mice frail at 32 months of age but compared this to 60+ years in humans, where 5-10% of 60-69-year-olds or 26-65% of 85+-year-olds are frail [26]. Another publication suggests that a 32-month-old mouse is equivalent to a 109-year-old human [35]. Kane et al. observed 16-44% of 23-month-old mice as frail depending on the FI index used, while humans aged 65+ years showed a 22-32% frailty range using comparable indexes [22]. Two FIs quantified different animals of the same age group as frail [22]. Furthermore, another study compared three FIs in a group of 24-month-old males and found inconsistency between which mice were frail [31].

Like the heterogeneity of mouse data, the lack of a universally agreed standard of human frailty scoring ensures that the reference ages for the percentage of the population identified as frail at a given age will also be inconsistent. The difficulty in making precise comparisons is evident, though the underlying fact of deficit accumulation increasing with age remains. Developing a simplified approach for FI calculations in murine models and humans is essential for standardizing assessments, improving reproducibility, and validating medical interventions for their translational potential for human aging and frailty management.

We strived to provide recommendations for implementing a FI in murine models with commonly available equipment and inform our analysis of the challenges in doing so. To this end, we scored our mice on the 8-item FI developed and implemented in the literature, as it suited the available equipment and allowed frailty to be measured as a gradient [15, 16]. To score the mice in our study, we utilized the reference values from these studies. It is clear from this implementation that the reference ranges in these studies are not consistent, and further work will be required to develop reproducible reference ranges. However, our data are underpowered and are intended for illustrative purposes only and should not be used to draw independent conclusions. As the reference values of one study had both sexes, and our cohort did too, we matched them by sex [16]. Notably, the other reference value cohort was female, and most of our subjects were male [15]. However, the 3-4-month-old mice in this study are younger than those used to establish the reference values [15, 16].

Sex as a biological variable in FIs is an important consideration, as there is a known difference between male and female frailty onset and progression. In humans, females show higher frailty index scores in all ages compared to males [39]. For aging studies in mice in this Perspective, males are predominantly used (10/18 studies) [17, 18, 20–24, 26, 31, 32] with both sexes (5/18 studies) [16, 19, 25, 27, 29] and females (3/18 studies) [15, 28, 30] used significantly less. One study measuring both sexes found males to have higher frailty scores in an Alzheimer’s model [29]. Another found that in C57BL/6 mice the frailty index implemented altered which sex had the higher frailty score [27]. Therefore, with the key role sex can play in frailty, it is preferred to separate the age groups by sex.

**Figure 1:**
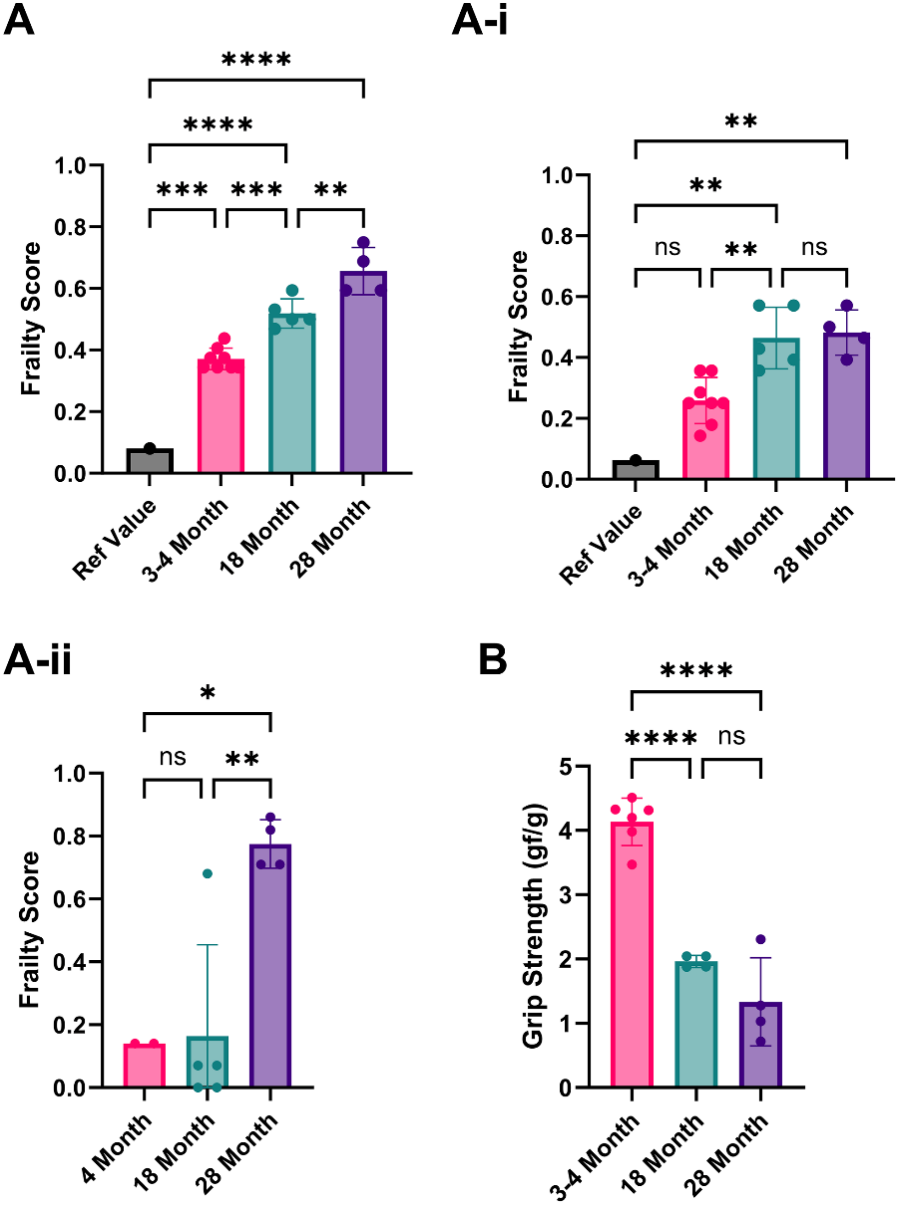
**Analysis of the frailty phenotype in 3-4, 18, and 28-month-old C57BL/6 mice.** (**A**) Frailty index scores implemented using Parks et al., 2012 reference values. (**A**–**i**) Frailty index scores implemented using Whitehead et al., 2014 reference values. (**A**–**ii**) Frailty index scores using our own 3–4-month-old mice as reference values. (**B**) Grip strength (average forelimb parallel bar score) is normalized to weight for 3-4, 18, and 28-month-old mice. P-values ≤ 0.05 (*), ≤ 0.01 (**), ≤ 0.001 (***), ≤ 0.0001 (****) were calculated using one-way ANOVA for three or more independent groups or unpaired t-tests for just two independent groups. Columns represent the mean with error bars ± SD.

Using the first set of published reference values, the 8-item FI yielded frailty scores of 0.37/1 for our 3-4-month-old mice, 0.52/1 for our 18-month-old mice, and 0.66/1 for our 28-month-old mice (Figure 1A) [16]. The second published reference values (averages of trial 1+2) yielded scores for the 3-4-month-old mice as 0.26/1, 18 months as 0.46/1, and 28 months as 0.48/1 frailty scores (Figure 1A–1i) [15]. In both reference sets, the frailty scores among the 3–4-month-old animals were notably high. The parameters responsible for scoring young mice as frail were the maximum distance post inactivity, meander, and movement duration, which affected this group's overall frailty score.

High scores in movement duration in our young mice may be due to differences in acclimatization protocols. Parks et al. allowed 5 days of testing in the arena, using the last 2 days for assessment [16]. We acclimatized the mice to the testing room but not the arena for an hour before data collection, as open field testing depends on the inherent explorative nature of mice. The meander can also be measured differently, either as a relative or absolute meander, using Ethovision video software analysis (version 17.0.1). It can also be calculated from either the body point or the head direction. The maximal distance post-inactivity could also benefit from further clarification on how it is precisely measured; for example, how inactivity was defined and what body point was measured.

Based on these potential sources of discrepancy, we recommend each lab use its own reference mice to limit variability. When we did this using our 3-month-old mice to establish the reference values, the overall frailty scores in mice aged 18-28 months were much lower than the reference values that were used from similarly aged mice from published literature (0.03/1 [16] and 0.22/1 [15], respectively (Figure 1Aii)). Most studies (12/18) have used C57BL/6 mice to assess new FIs or modify existing FIs. Therefore, if using a different mouse strain or a transgenic or disease model, identifying the baseline of a lab’s rodent population is even more critical. We recommend using each mouse as its reference point for longitudinal studies, strengthening the analysis without increasing the workload, as the cost is often a significant factor for *in vivo* studies involving aged subjects, especially longitudinal studies. A common strategy for anti-aging interventions is to collect baseline data before and after treatment.

One challenge when creating reference values from a group of young mice is inherent variation within the group. If the reference group variation is significant, some of the frailty parameters in test mice may not reach a score of 1. One strategy to circumvent this is to reduce diversity in the reference value group. For example, Antoch et al. excluded animals if their scores exceeded the mean by more than one SD [25]. However, inclusion or exclusion of the outliers in small sample sizes risks creating non-representative values. Using each mouse as its reference point can also circumvent this potential issue. Similarly, to ascertain if a treatment can improve frailty we suggest scoring the same mice before treatment and utilizing that as individual reference values, instead of comparing the FIs of an aged, treated group with young, untreated mice as commonly done in rodent aging studies.

Similar to 4-5 item FIs, we included grip strength to characterize the physical health of the mice further. Due to equipment limitations, we were unable to fully implement the 4-5 item FI, which required an inverted cling-grip test, a rotarod, and voluntary wheel running cages. In grip strength scores normalized by weight, the 3- to 4-month-old mice had the highest average score, with 18- and 28-month-old mice having lower scores accordingly. However, the difference between 18 and 28 months was not statistically significant due to a single animal’s exceptionally high score (Figure 1B). Using individual mice as their comparators in longitudinal assessments could help identify any increases or decreases in performance. Normalization by weight is imperfect because it does not account for the changes in muscle mass and fat between young and old mice, as older mice tend to gain fat and lose muscle mass. To address this, body composition measurements would be preferable, although they require specialized and expensive equipment.

One limitation of this study is the low number of animals per group, which limits the results for animals of a given age, as some recommend a minimum of 20 animals per sex to measure physiological changes. However, this Perspective may be helpful in frailty-scoring selections for small-scale studies with limited subjects.

Open-field testing (OFT) or automated video testing simplifies frailty measurements when the methods are clearly defined and well-documented. Furthermore, it eliminates errors introduced by limited inter-rater reliability, a well-recognized problem in clinical observation FIs, as user input is minimal [18, 20]. To determine the most significant factors from OFT to include, an independent study is necessary to identify which parameters best predict mortality. For instance, investigators could make periodic measurements of the various OFT parameters (e.g., every 3 months) with a significant sample size until death, and then assess each factor to determine its weight in predicting mortality compared to an established FI.

The reason for this extensive independent study is the components of the current indexes. For instance, 8 item FIs include the total distance (cm), velocity (cm/s), and movement duration (s and %). Therefore, 4/8 factors measure closely related physiological traits, giving movement great weight in this index. Although walking speed is a well-established mortality indicator and predictor of surgical outcomes in humans, assigning movement-related variables half the overall score in a rodent FI will likely overweight the index [6, 7]. Principal component analysis should be applied to reduce the number of variables and choose those that are both highly predictive of mortality and (amongst those that fall within the same principal component) most convenient for implementation. One study prioritized a diversity of health-related physiological systems over a single key physiological trait, alongside quantitative parameters without visual scoring, while maintaining minimal invasiveness [25]. For future additions to FIs, one study measured gait speed in the cage and on the wheel in C57BL/6 mice and found it correlated with age and the manual frailty index [32].

Further development of frailty indices could improve their accuracy. Quantitative and automated measures would help further reduce inter-rater reliability concerns and experimenter bias, and in turn lab-to-lab variation. Measurements such as bone density, measured non-invasively by micro-CT, have been proven to show differences in murine age, especially in cranio-facial bones [40–42]. However, this technique comes with a significant cost. Changes in eating behaviour, such as food dropping, reduced consumption, or altered eating patterns, serve as another quantitative measure observed in aging mice for future frailty indices [43, 44].

Aging also affects the circadian rhythm and feeding patterns in mice, as caloric restriction and feeding during the active phase of the circadian rhythm resulted in extended healthspan and lifespan [45]. One study in this Perspective identified an age-related change in the circadian distribution of wheel running, suggesting the inclusion of the circadian rhythm in frailty indices is warranted [32]. Evidence in humans corroborates the significant changes in the circadian rhythm with age, potentially offering additional translational evidence for future frailty indices [46]. To assess the age-related decline in cognitive function, namely spatial learning and memory, a Barnes-Maze test could be implemented. One group developed a cognitive frailty index (CoFI) to assess various parameters of the Barnes-Maze test in over 400 C57BL/6 mice [47]. While no sex-related differences were observed, an increase in CoFI scores with advancing age and a strong association with mortality were evident [45, 32, 46].

Another aspect is olfactory tests, which measure the loss of smell evident in aged C57BL/6 mice, where the loss of odor discrimination was among the earliest biomarkers compared to cognitive and motor function tests [48]. Olfactory tests have been included in a mouse Social Frailty Index (mSFI), which also analyses urine marking, social interactions, and nest building [49]. Interestingly, sex differences were observed, with females exhibiting lower mSFI scores compared to males, which correlates with data from physical indices. The application of a physical and cognitive/social frailty index could provide greater insight into the holistic effect of any anti-aging intervention.

## CONCLUSION

Studies discussed in this Perspective offer a variety of approaches to measuring frailty. We recommend that investigators carefully consider what aspects of frailty to include in their analyses instead of fully adopting the published scoring systems. It is preferable to avoid including and giving equal weight to multiple parameters that measure closely related physiological traits, such as multiple parameters related to movement. In addition, it is best to use automated measurements where possible to avoid experimenter bias and inter-rater variations. As there is substantial variation even between mice of the same strain, it is optimal to measure baseline frailty in each animal and to track changes over time in response to treatment and during physiological aging, especially in a small sample size. Doing so accounts for variability in baseline values. It increases sensitivity in detecting subsequent changes while reducing the chances of false positives, resulting in a more reliable measurement of physical health in aging rodents and the effects of longevity therapeutic candidates.

## Supplementary Material

Supplementary Table 1
