# Supplementary material for: Analysis of the current state of frailty indexes and their implementation for aging intervention studies
Source: Aging (Albany NY). 2025 Aug 26;17(9):2241–51. doi: 10.18632/aging.206307 (PMC12517216; doi:10.18632/aging.206307)
Supplement: Supplementary Table 1 [file aging-17-9-206307-s001.docx]

**Supplementary Table 1. Summary of frailty index studies.**

| **Author**  **Year (PMID)** | **Breed**  **Age**  **Sex (n-number)** | **Frailty Index Measurements** | **Frailty definition/cutoff point** | **Key Takeaways** |
| --- | --- | --- | --- | --- |
| **Parks et al., 2012**^15^  **(22021390)** | C57BL/6  13.5 and 30.3 months old  Male (n=230) and Female (n=191) | 8 Item FI:  Total distance, max distance post inactivity, total duration movement, % time spent moving, meander (change direction), velocity, rearing frequency, weight  31 Item FI:  Activity levels, Hemodynamic measures, body composition  Basic metabolic status  Measured every day for 5 days. | 8+31 Item FI:  < 1SD = 0  ± 1SD = 0.25  ± 2SD = 0.5  ± 3SD = 0.75  > 3SD = 1  Sum divided by total parameters | 8 Item FI:  FI scores (13-30 months) of 0.07 - 0.73 in males, and 0.08 - 0.52 in females.  0.73 score is close to the 99% limit for FI in 65+ year-old humans (0.7).  No difference in mortality between males and females. |
| **Liu et al., 2014**^16^  **(24336799)** | C57BL/6  27-28-months  Male (n=11) | 4 Item FI:  Grip Strength, Walking Speed, Physical Activity, Endurance  One-time measurement. | 1.5 SD below the mean (lowest 7%)  ≥3 criteria = frail, 2 = mildly frail, ≤1 = non-frail | Matches Fried definition of human frailty.  9% of mice exhibited as frail, consistent with humans at ‘same age’ (80 yrs).  Voluntary wheel running didn’t identify any as frail. |
| **Whitehead et al., 2014**^17^  **(24051346)** | C57BL/6  5, 19, and 28-month-old female mice (n = 5, 4, 5) | 8 Item FI:  Total distance, max distance post inactivity, total duration movement, % time spent moving, meander (change direction), velocity, rearing frequency, weight  31 Item FI:  Integument, Physical, Auditory, Nasal/Ocular, Digestive/Urogenital, Discomfort, Other  Measured twice 10 days apart. | 8 Item FI:  < 1SD = 0  ± 1SD = 0.25  ± 2SD = 0.5  ± 3SD = 0.75  > 3SD = 1  Sum divided by total parameters  31 Item FI:  Absent = 0  Mild = 0.5  Severe = 1.0 | 8 Item FI:  FI scores (5-19-28 months) of 0.06, 0.36, 0.38.  31 Item FI:  FI scores (5-19-28 months) of 0.02, 0.12, 0.33.  Deficit accumulation (natural log FI Vs age) showed 31 items FI as 0.038 compared to 0.034 in humans.  Normalised to 90% mortality, frailty in humans is closer when using 31 items rather than 8 items in mice. |
| **Feridooni et al., 2014**^18^  **(25205762)** | C57BL/6 343-430-day old male mice (n = 233 total – split into 3 groups) | 31 Item FI:  Whitehead et al., 2014 with some modifications in assessment  Measured between 10:00-14:00 each day of testing.  Group 1 = 343-357 days old  Group 2 = 370- 377 days old  Group 3 = 387-430 days old | Absent = 0  Mild = 0.5  Severe = 1.0    Bodyweight and surface temp:  < 1SD = 0  ± 1SD = 0.25  ± 2SD = 0.5  ± 3SD = 0.75  > 3SD = 1  Sum divided by total parameters | Focus on testing inter-rater reliability which improved over time: Group 1 - 11 scores differed. Group 2 - 6 scores differed. Group 3 - 2 scores differences.  FI scores of 0.213 and 0.212 regardless of the rater.  Group 2+3 FI is significantly higher than Group 1. |
| **Yorke et al., 2017**^19^  **(28158648)** | F344 Fischer 6-9- and 13-21-month-old male rats (n = 12, 41) | 27 Item FI:  Whitehead et al., 2014 without loss of fur colour, whiskers, tail stiffening, forelimb grip strength, vestibular disturbance, vision loss, menace reflex, nasal discharge, grimace scale.  Replaced with unusual sounds, food intake, jaundice, head tilt, chromodacryorrhea.  Measured every 3-4 months up to 21 months old. | Absent = 0  Mild = 0.5  Severe = 1.0  Bodyweight, food intake, surface temp:  < 1SD = 0  ± 1SD = 0.25  ± 2SD = 0.5  ± 3SD = 0.75  > 3SD = 1  Sum divided by total parameters | FI scores in 13, 17, 21 months were 0.06, 0.13, 0.21.  Normalised to 90% in mice and human data. Deficit accumulation was 0.045 in rats, 0.038 in mice, and 0.034 in humans. Due to the higher rate deficit accumulation, the cutoff point may need to be lower.  The oldest rats were 5 months below 50% mortality. |
| **Rockwood et al., 2017**^17^  **(28220898)** | C57BL/6 3–4-week-old male mice (n = 251) | 31 Item FI:  Used the Feridooni et al., 2014 modified version of Whitehead et al., 2014  Measured throughout lifetime at 1, 6, 12, 18 months of age. | Absent = 0  Mild = 0.5  Severe = 1.0  Bodyweight and surface temp:  < 1SD = 0  ± 1SD = 0.25  ± 2SD = 0.5  ± 3SD = 0.75  > 3SD = 1  Sum divided by total parameters | Deficit accumulation in mice 0.036, and 0.029 in humans.  The submaximal frailty limit is 0.44 in mice and 0.54 in humans.  Relationship to mortality (mice = 1.15, human = 1.05)  Human data was from both sexes, mice only male. |
| **Kane et al., 2017**^20^  **(28549083)** | C57BL/6 101-week-old male mice (n = 36) | 5 Item FI:  Modified Liu et al., 2013.  Grip strength, activity, walking speed, endurance, weight loss.  The front paw hang instead of four paws. Open-field assessment instead of voluntary wheel running. Rotarod but no training protocol. Endurance is still the wire hang and rotarod.  Also uses Whitehead et al., 2014 31 points.  One time measurement | Liu: 0.8 SD below cohort mean (lowest 20%)  Whitehead: <0.18 = nonfrail, ≥0.18 = frail. Upper 50% confidence score of mean FI score in cohort. | Modified FI identified 16.6% of mice as frail, whereas the 31 item FI identified 44.4% of mice as frail. In humans aged 70-85 with  6-16% of humans aged 70-85 years were frail using phenotype, or 22-32% with clinical FI but 65+ years old.  Frailty indexes only had 50% agreement in identifying mice as frail. |
| **Miller et al., 2017**^21^  **(28329224)** | F344 17-months-old male rats (n = 133) | 4 Item FI:  Modified Liu et al., 2013  Grip strength, walking speed, physical activity, endurance.  Forelimb wire suspension instead of inverted cling grip. Open field instead of voluntary wheel running. Inclined screen test instead of (grip test + rotarod) /2.  One time measurement. | In large studies: Frail = lowest 20% performance in ¾ tests.  Mildly frail = lowest 20% in 2/4 tests.  Non-frail = lowest 20% in ¼ of tests.    For comparison: 1.5SD below mean in ¾ tests were frail.  1.5SD below mean in 2/4 tests were mildly frail.  1.5SD below mean in ¼ tests were nonfrail. | Frailty assessment in rats identified 17.29% as mildly frail, and 2.26% as frail.    Measuring 100-day survival, mildly frail and frail rats were 3.8 and 27.5 times more likely to die than nonfrail rats. |
| **Gomez-Cabrera et al., 2017**^22^  **(28329258)** | C57BL/6 3-month-old male mice (n = 120 in total – split in 2 groups)  Sedentary vs. spontaneous exercise | 5 Item FI:  Valencia Score; made of weight loss, grip strength, endurance, slowness, and activity level.  Includes weight, (horizontal fore-limb grip strength), treadmill, and tightrope test.  Tested at ages 17, 20, 23, 26, 28 months. | 20^th^ Percentile as the cutoff point which meant fail.  Bodyweight was more than 5% loss.  The total number of tests failed, divided by the total number of tests performed, was a %. | Compared sedentary (s) and active lifestyles (a) on mice frailty and age.  At 17 months, s = 34%, a = 2% frail.  At 23 months, s = 35%, a = 8% frail.  At 28 months, s = 83%, a = 63.  Wheel running improved frailty score as the mice aged. |
| **Antoch et al., 2017**^23^ **(28325885)** | NIH Swiss 6–8-week-old male and female mice (n = 10-14 per age group) | 29 Item FI:  Adapted from Parks et al., 2012  Physical fitness, cardiovascular system, total blood cell composition, plasma concentration  Tested at ages 6, 12, 18, 24 months. | < 1SD = 0  ± 1SD = 0.25  ± 2SD = 0.5  ± 3SD = 0.75  > 3SD = 1  Sum divided by total parameters | FI scores increase with age in NIH Swiss mice.  High fat diet (HFD) decreases lifespan and FI scores (only in males).  FI score shows difference when treatment is used in males. |
| **Baumann et al., 2018**^24^ **(30562163)** | C57BL/6 14-month-old male mice (n = 29) | 5 Item FI:  Modified from Liu et al., 2013.  Body weight, walking speed, strength, endurance, physical activity.  Tested every 3 months until death. | Bottom 20% of the cohort unless it is weight and that is top 20%. | Every mouse is frail by 32 months. Human frailty is 5-10% at 60-69 years of age.  A strong link between frailty and mortality at 23 months plus. |
| **Kane et al., 2019**^25^  **(29788087)** | C57BL/6 17–22-month-old male and female mice (n = 37, 17F) (n = 34, 17M) (n = 17, 22F) (n = 13, 22M) | Use three indexes:  31-point FI-Clinical:  From Whitehead et al., 2014.  23 Item FI-Lab:  Blood pressure, basic metabolic status, echocardiography.  Combination of FI clinical and FI lab scores.  Tested at two different ages (17 months and 22.5 months). | Absent = 0  Mild = 0.5  Severe = 1.0    Weight and surface temp:  < 1SD = 0  ± 1SD = 0.25  ± 2SD = 0.5  ± 3SD = 0.75  > 3SD = 1  Sum divided by total parameters.  For FI-Lab: Values within 1.5SD scored 0 and those greater than 1.5SD scored 1 as a deficit. | FI-Clinical scores higher in females, but FI-Lab scores higher in males, if FI scores combined no sex differences.  Mice frailty didn’t correlate between FI-Lab and FI-clinical scores whereas in humans they do.  Found a positive correlation between all 3 FI indexes and pro-inflammatory cytokines. |
| **Kwak et al., 2020**^26^  **(30958526)** | C57BL/6 17-month-old female mice (n = 27) | 5 Item FI:  Used Baumann et al., 2018.  Tested every 3 months from 17m until death. | Bottom 20% of the cohort unless it is weight and that is top 20%. | At 17 months, 1/27 were frail, 11.1% were pre-frail. At 26 months, 36.9% were pre-frail. At 32 months, 66.7% were frail.  Frail and pre-frail mice lived to 26 months; non-frail lived to 29 months. |
| **Scheuren et al., 2020**^27^ **(32966306)** | Accelerated aging model (PolgA) 34-week-old female mice (n = 12 per age group)  The average lifespan ranges from 36-59 weeks. | 31 Item FI:  From Whitehead et al., 2014.  Tested at 34, 38, 40, 44 weeks old. | Weight and body temperature:  < 1SD = 0  ± 1SD = 0.25  ± 2SD = 0.5  ± 3SD = 0.75  > 3SD = 1    Rest of index:  Absent = 0  Mild = 0.5  Severe = 1.0 | Accelerated aging exhibited higher FI scores compared to wildtype. Differences observed only after week 38.  No difference in bodyweight between WT and PolgA. |
| **Todorovic et al., 2020**^28^ **(31837369)** | 5xFAD Model of Alzheimer’s 3 and 11-month-old male and female mice  (n = 6, 3M)  (n = 6, 3F)  (n = 8, 11M)  (n = 10, 11F)    Average lifespan is 15-months. | 8 Item FI:  Modified from Liu et al., 2013 Miller et al., 2017.  Weight, open field testing, grip strength, tight-rope test.  24 Item FI:  Modified from Yorke et al., 2017 | 8 Item FI:  Bottom 20% Cutoff point, bodyweight 5%.  Total number of tests failed/ total tests performed as a %.  24 Item FI:  Absent = 0  Mild = 0.5  Severe = 1.0  The sum of values is divided by the number of parameters. | 8 Item FI:  12% young male mice and 46% old male mice were frail.  20% young female mice and 36% old female mice were frail (not stat sig).  Females less frail than males with age.  24 Item FI:  Male almost 20 times higher FI score, smaller but significant female FI score. |
| **Ribeiro et al., 2022**^29^ **(34181005)** | EAE (MS Model) 3-, 6-, and 12-month-old female mice (n = 5 per group) | 34 Item FI:  Modified from Whitehead et al., 2014.  Physical condition, neuromusloskeletal reflex, paralysis, strength, ataxia, integument, grooming, auditory/ocular/nasal, digestive, urogenital, respiratory, discomfort. | 24 Item FI:  Absent = 0  Mild = 0.5  Severe = 1.0  Weight and surface temp:  < 1SD = 0  ± 1SD = 0.25  ± 2SD = 0.5  ± 3SD = 0.75  > 3SD = 1  The sum of values is divided by the number of parameters. | After induction of MS in mice, frailty scores of 0.36 and 0.43 were seen in 3- and 6-month-old mice respectively.  EAE mice higher frailty score than age matched control. |
| **Mach et al., 2022**^30^  **(35032570)** | C57BL/6 24-month-old male (n = 23) | Used three FI to compare:  Liu et al., 2013  Kane et al., 2017  Baumann et al., 2018  Also measured 31-point:  Whitehead et al., 2014  Tested every 3 months (12, 15, 18, 21, 24). | For Liu et al., 1.5SD.  For Kane et al., 0.8SD.  Baumann et al., Lowest 20^th^ Percentile. | All mouse frailty tools quantified different mice as frail.  Frailty prevalence at 24 months:  Kane et al., 8.3% now 13.9% then. Baumann et al., 12.5% now 10.7% then.  Liu et al., not specified as mice younger than Liu original study. |
| **Ruby et al., 2023**^31^ **(36708182)** | C57BL/6 3-month-old male mice (n = 31) | 29 Item FI:  Modified from Whitehead et al., 2014, where bodyweight and temperature were omitted due to population-specific statistics.  Tested at 7, 19, 35 months.  Between 9-11 am each day. | Absent = 0  Mild = 0.5  Severe = 1.0  The sum of values is divided by the number of parameters. | FI using home-cage automated analysis.  Digital FI (DFI)scores correlate with manual FI (MFI) and chronological age.  DFI and age correlation = 0.76.  DFI and MFI correlation = 0.48. |
